# The Dopamine D5 receptor contributes to activation of cholinergic interneurons during L-DOPA induced dyskinesia

**DOI:** 10.1038/s41598-020-59011-5

**Published:** 2020-02-13

**Authors:** Julia Castello, Marisol Cortés, Lauren Malave, Andreas Kottmann, David R. Sibley, Eitan Friedman, Heike Rebholz

**Affiliations:** 1 Department of Molecular, Cellular & Biomedical Sciences, CUNY School of Medicine, New York, NY USA; 2 Ph.D. Programs in Biochemistry and Biology, The Graduate Center, CUNY, New York, USA; 3 Molecular Neuropharmacology Section, National Institute of Neurologic Disorders and Stroke, Intramural Research Program, National Institutes of Health, Bethesda, Maryland USA; 4 Institut de Psychiatrie et Neurosciences de Paris (IPNP), UMR_S1266, INSERM, Université de Paris, 102-108 rue de la Santé, F-75014 Paris, France; 5 GHU PARIS psychiatrie et neurosciences, Paris, France; 6 Danube Private University (DPU), Krems, Austria

**Keywords:** Parkinson's disease, Molecular neuroscience

## Abstract

The dopamine D5 receptor (D5R) is a Gα_s_-coupled dopamine receptor belonging to the dopamine D1-like receptor family. Together with the dopamine D2 receptor it is highly expressed in striatal cholinergic interneurons and therefore is poised to be a positive regulator of cholinergic activity in response to L-DOPA in the dopamine-depleted parkinsonian brain. Tonically active cholinergic interneurons become dysregulated during chronic L-DOPA administration and participate in the expression of L-DOPA induced dyskinesia. The molecular mechanisms involved in this process have not been elucidated, however a correlation between dyskinesia severity and pERK expression in cholinergic cells has been described. To better understand the function of the D5 receptor and how it affects cholinergic interneurons in L-DOPA induced dyskinesia, we used D5R knockout mice that were rendered parkinsonian by unilateral 6-OHDA injection. In the KO mice, expression of pERK was strongly reduced indicating that activation of these cells is at least in part driven by the D5 receptor. Similarly, pS6, another marker for the activity status of cholinergic interneurons was also reduced. However, mice lacking D5R exhibited slightly worsened locomotor performance in response to L-DOPA and enhanced LID scores. Our findings suggest that D5R can modulate L-DOPA induced dyskinesia and is a critical activator of CINs via pERK and pS6.

## Introduction

Parkinson’s disease (PD) is the second most prevalent neurodegenerative disease, it affects up to 4% of people aged 65 years or more^1^. Hallmarks of the disorder are motor symptoms such as tremor, rigidity, bradykinesia and gait disturbances^2^, which are a result of the selective degeneration of dopaminergic neurons within the substantia nigra pars compacta and thus not by but of dopamine depletion^3^. Up to today, L-DOPA treatment remains the gold standard treatment for PD. In the majority of patients, the efficacy of L-DOPA is reduced and debilitating involuntary movements, termed L-DOPA-induced dyskinesia (LID), develop during chronic L-DOPA treatment over several years^4^. Various pharmacological tools are currently investigated with the aim to mitigate these complications.

The striatum is comprised mainly by two types of GABAergic projection neurons (SPNs) that are categorized based on their predominant expression of either D1- or D2 receptors, together making up over 95% of all striatal neurons^5^. The spiny neurons that project directly to the basal ganglia output nuclei are termed ‘direct pathway’ or dSPNs and express D1 receptor (D1R), that signals via the G proteins Gα_s_/Gα_olf_ to PKA and enhances cAMP production. The other class of projection neurons is termed ‘indirect pathway’ or iSPNs, and they express the inhibitory, Gα_i/o_-coupled D2 receptor (D2R)^6^, leading to less cAMP being produced. The balance between the two pathways is crucial for voluntary movement control and is disturbed in PD. In addition, cholinergic interneurons (CINs), which represent 1–3% of striatal neurons but are extensively arborized, gate striatal output by modulating SPN activity and DA release into the striatum^7–9^.

The D5 receptor belongs to the D1-type family of dopamine receptors that are coupled via Gα_s_/Gα_olf_ and initiate cAMP/PKA signaling^10^. Compared to the D1 receptor, in most regions of the rodent and human brain much less D5R is found to be expressed^11^. However, the D5R has a 10-fold higher affinity for dopamine and higher constitutive activity^12^. On a cellular level, the expression ratio of these receptors differs in cell types: The D1 receptor is present in all direct pathway spiny projection neurons (dSPNs) but the D5R is not expressed. All indirect pathway spiny projection neurons (iSPNs) express D2 type receptors, but only 11–20% express either D1 or D5R^13^. Interestingly, the D5 receptor is expressed in 88% of cholinergic interneurons (CINs) while only 17% of them express the D1 receptor and none express D3 or D4 receptor^13,14^. Furthermore, CINs express twice as much D5R compared to SPNs^15^.

Due the lack of pharmacological tools that target D1R and D5R specifically, the study of the biological roles of the two receptors has proven difficult *in vivo*. Hence, the generation of KO mice where D1 or D5 receptors are ablated has been helpful. Drd5^(−/−)^ mice (which in the remainder of the text will be termed D5 KO), are generally viable, healthy and fertile^16,17^. These mice do not exhibit any compensatory up- or down- regulation of other dopamine receptor subtypes^16^. Locomotor activity is normal, as tested by open field recording and the rotarod test^17^. D1 KO mice lack a response to acute cocaine, but retain the sensitization to chronic cocaine treatment^18,19^. In contrast, D5 KO mice respond normally to acute and chronic cocaine and present normal conditioned place preference in response to cocaine^19^. Interestingly, D5 KO are more sensitive to a methamphetamine, an effect that is mediated by the DA transporter^20^. Startle response and pre-pulse inhibition are normal in these mice. Memory assessing paradigms (Morris water maze, and cued and contextual fear conditioning) are also not altered^17,21^, however, when probed more thoroughly, such as for example in a paired-trial T-maze test and a temporal order object recognition task, the KO mice exhibited reduced performance^22^.

In the prefrontal cortex, a distinguishable function for the D5 over the D1 receptor has been described since D1-type agonist stimulation of the BDNF-TrkB-Akt signaling pathway is still present in D1 KO mice, but abolished in D5 KO mice^23^.

L-DOPA induced dyskinesia is thought to be mainly driven by hypersensitivity of D1-mediated signaling in dSPN where L-DOPA leads to activation of the ERK signaling pathway^24,25^. The numbers of pERK-positive direct pathway SPNs correlate with LID scores^26^. It was also shown that ERK-dependent plasticity mediates the aberrant response to chronic L-DOPA^27^. In addition, pharmacological ERK inhibition alleviates LID^28,29^.

Recent studies suggest that striatal cholinergic interneurons are involved in the development of LID^30–32^. CIN activity and acetylcholine release are regulated by muscarinic M1, M2 and M4 auto-receptors and D2 and D5 receptors. Thus, L-DOPA must exercise its effect on CINs through the ratio of expression of these two DA receptors, which act in opposing ways, inhibiting and activating adenylyl cyclase, respectively. Since ERK was shown to be activated during chronic L-DOPA treatment in CINs, we hypothesize that phosphorylation of ERK1/2 could be mediated through the D5 receptor, possibly in a manner analogous to D1 receptor mediated activation of pERK, involving crosstalk with the NMDA receptor^33^. The use of D5 KO mice enabled us to examine the role of the D5 receptor in the effects of 6-OHDA lesion and L-DOPA treatment, to observe changes in LID onset/severity and in biochemical correlates of neuronal activity and LID.

## Results

### Altered dopamine D5 receptor expression in the dopamine-depleted brain

**Figure 1:**
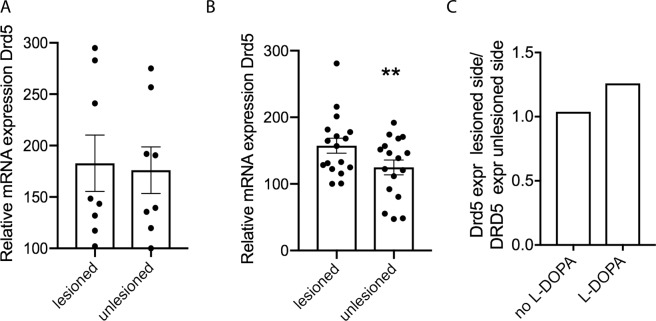
Expression of Drd5 is changed in the DA depleted striatum. qPCR was performed on striatal tissue derived from both hemispheres of wildtype C57Bl/6 mice that had been unilaterally 6-OHDA lesioned 4 weeks prior (**A**) (N = 8), or that had been lesioned and treated with L-DOPA (3 mg/kg; ip) daily for 7 weeks (**B**) (N = 17). Normalization was performed with GAPDH. Graphs show individual and mean values +/− SEM. Statistical analysis was performed using Wilcoxon Matched Pairs t-test (**P < 0.01).

Since no data exist about the involvement of the D5 receptor in PD and LID, we first wanted to investigate if its striatal expression is altered in response to dopamine depletion and to chronic L-DOPA in the unilateral 6-OHDA PD model. Using qPCR, and comparing lesioned with unlesioned hemispheres, we found that 4 weeks post-lesion the expression of the dopamine D5 receptor transcript in striatal tissue was similar in both hemispheres (Fig. 1A), however, in response to chronic L-DOPA treatment (3 mg/kg for 7 weeks), Drd5 mRNA was significantly higher in the lesioned hemisphere compared to the unlesioned side of WT mice (p < 0.05) (Fig. 1B). The ratio of Drd5 expression lesioned/unlesioned hemisphere in the chronically L-DOPA treated mice is 1.26 whereas it is 1.04 in the lesioned mice and non-treated mice (Fig. 1C).

### Motor performance is affected in dyskinetic D5 KO mice

**Figure 2:**
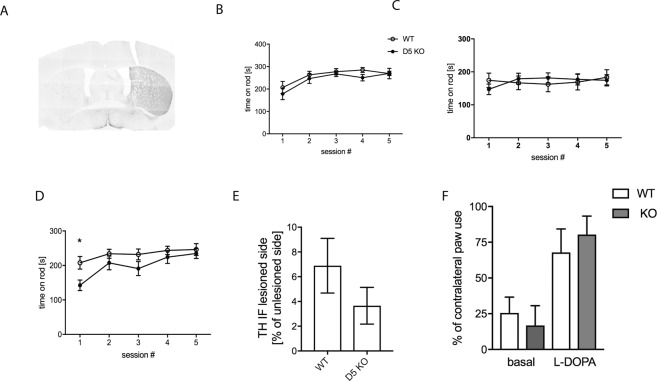
Motor behavior of 6-OHDA lesioned D5 KO mice. Representative image of immunohistochemical analysis using anti-TH antibody of a coronal slice from 6-OHDA lesioned D5 KO mouse (**A**). Quantification derived from ImageJ density measurements is shown (**E**). The rotarod test was performed with the same animals before lesion (**B**), 3 weeks post lesion (**C**) and after 14 days of L-DOPA treatment (**D**) (N = 15 before and 12/10 after lesion for both WT/KO). The anti-akinetic response to L-DOPA was measured in the cylinder test (**F**). Statistical analysis was performed using two-way ANOVA: Effect of genotype: F_(1,100)_ = 9.72, p = 0.002. Effect of time: F_(4,100)_ = 4.79, p = 0.001. Post-hoc comparisons (Sidak) (*p < 0.05). At each trial days mice were subjected to 5 sessions spaced 20 minutes apart. For (**F**), the test was administered starting at 90 min post L-DOPA injections. Graphs show mean values +/− SEM. Statistical analysis was performed using 2-way ANOVA and Sidak’s multiple comparisons post-test (*P < 0.05).

We next wanted to determine whether motor skills are affected in the D5 KO mice. It has been shown by others that D5 KO mice do not exhibit any deficiency in motor activity and/or exploration^17^. We could confirm this finding in the rotarod test since neither basal performance nor learning over the course of five consecutive trials (spaced by 20 minutes) were affected in the KO mice (Fig. 2B). After recovery from unilateral 6-OHDA lesion, KO and WT mice also performed similarly in the rotarod test (Fig. 2C), however when tested after two weeks of L-DOPA treatment (3 mg/kg) KO mice performed significantly less well when tested 90 min after the last injection (Fig. 2D) (2-way ANOVA, Sidak’s post-test, p < 0.05). As described by others, it was necessary to test the mice at 90 min after the last injection since animals are unable to hold on to the rod during L-DOPA peak time^34^. As shown in Fig. 2D, at the time points of 110 min post-L-DOPA or later, this difference in motor performance has disappeared, most probably because the effect of L-DOPA has worn off.

We needed to exclude the possibility that differences in susceptibility to the neurotoxin could have resulted in different extents of lesion and responsiveness to L-DOPA in KO versus WT mice. We determined the extent of lesion by quantifying the TH signal from coronal brain slices processed by immunohistochemistry. Reduction in TH signal was similar across both genotypes indicating that both were similarly affected by the toxin. The average remaining TH signal was 6.8 +/− 2.2% for WT and 3.7 +/− 1.5% for the D5 KO (Fig. 2A,E) in the dorsolateral striatum. For the same purpose, mice were also tested in the cylinder test. The extent of contralateral paw use was not significantly different between the genotypes, both basally as well as after L-DOPA administration (Fig. 2F).

### D5 KO mice exhibit enhanced dyskinesia

**Figure 3:**
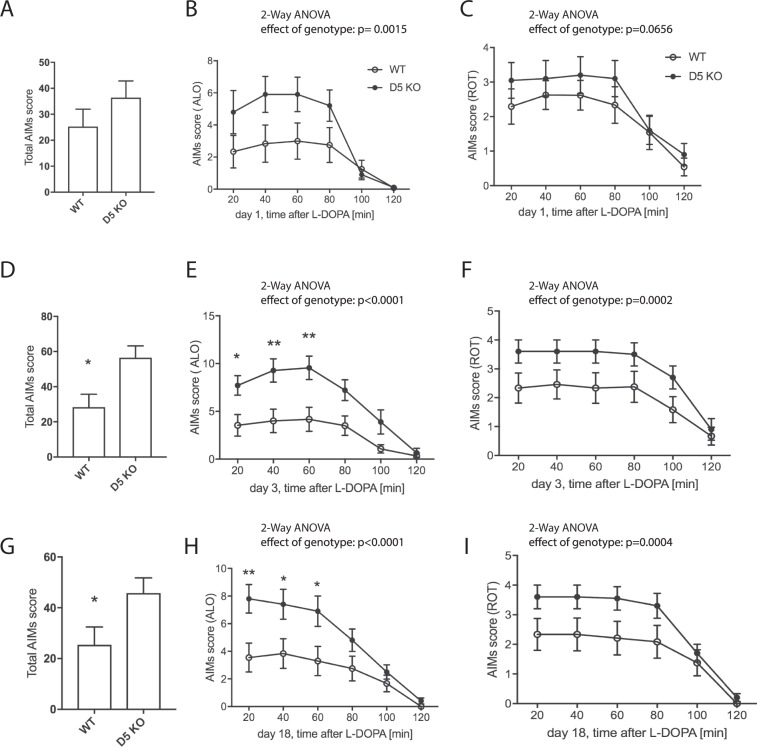
LID assessment of D5 KO mice. Mice were treated with L-DOPA/Benserazide (L-DOPA 3 mg/kg, Benserazide 10 mg/kg, i.p.) daily and AIMs determined starting at 20 min post injection. Total AIMs scores (**A**,**D**,**G**) ALO score (**A**,**E**), LOC score (**B**,**F**) and were determined and plotted as summarized score or as time course at 20 min intervals for 2 hours after L-DOPA injection (N = 10–12). SKF83959 (0.2 mg/kg, i.p.) was injected instead of L-DOPA and total AIMs, ALO and ROT scores assessed (**G**–**I**), (N = 6). Graphs show mean values +/− SEM. Statistical analysis was performed using Student’s t-test (A (p > 0.05), D, G (p < 0.05)) and 2-way ANOVA (Effect of genotype (**B**): F(1, 120) = 10.6, p = 0.0015; (**C**): F(1, 120) = 3.45, p > 0.05; (**E**): F(1, 120) = 41.5, p < 0.0001; (**F**): F(1, 120) = 15.26, p = 0.0002; (**H**): F(1, 120) = 23.58, p < 0.0001; (**I**): F(1, 120) = 13.4, p = 0.0004). Sidak’s multiple comparisons post-test (**P < 0.01;*P < 0.05).

To induce dyskinesia, we injected mice with L-DOPA/Benserazide (3 mg/kg/10 mg/kg) and assessed dyskinetic behavior. Acute dosage with L-DOPA has been shown to lead to low level dyskinesia^35^, which we also observed. On day 1 of treatment, mice presented ALO (axial, limb and orofacial) dyskinesia. While there was no significant difference between genotypes in the total AIM and the rotational score (Fig. 3A,C), the ALO score showed an effect of genotype, with D5 KO exhibiting enhanced ALO dyskinesia (p < 0.01). We next assessed LID on day 3, when the difference between genotypes was more pronounced: The KO mice showed enhanced total AIMs (Fig. 3D, p < 0.05), ALO AIMs (Fig. 3E, p < 0.0001), and, albeit to a lesser extent, rotational scores (Fig. 3F, p < 0.001). Similar results were obtained on day 18 of L-DOPA treatment (Fig. 3G–I).

We also tested whether a lower dose of L-DOPA would also display the difference in LID scores between the genotypes. A dose of 1 mg/kg to dyskinetic animals resulted in enhanced total dyskinesia (Suppl. Fig. 1A, effect of genotype p < 0.05) and ALO (Suppl. Fig. 1C, effect of genotype p < 0.001) and slightly enhanced ROT scores in the D5 KO (Suppl. Fig. 1F, effect of genotype, p < 0.01).

### Activity markers are significantly altered in CINs in dyskinetic D5 KO mice

**Figure 4:**
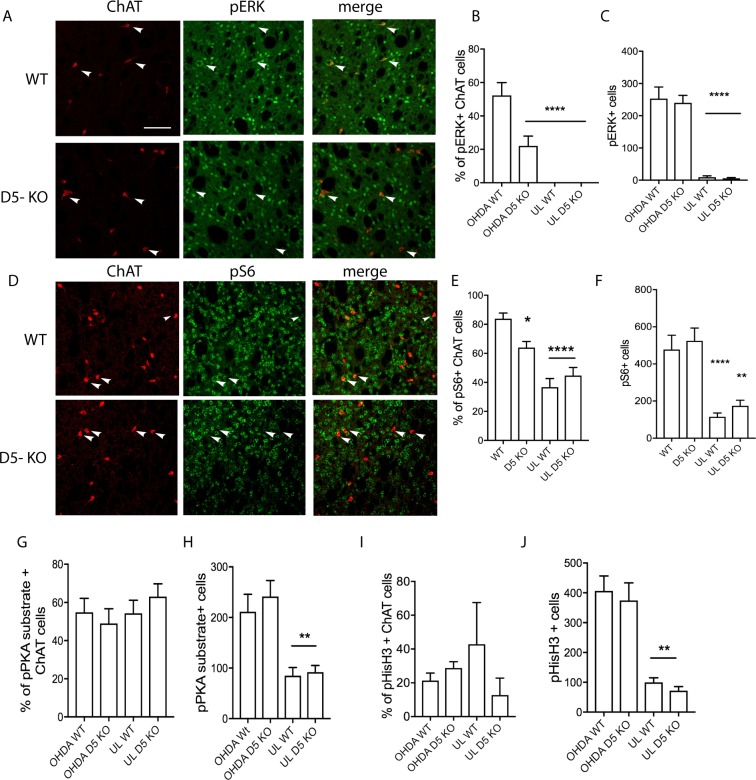
Markers of CIN activation are altered in Drd5-KO mice. Immunohistochemical analysis of coronal striatal slices from WT and Drd5-KO mice and quantification thereof after chronic L-DOPA/Benserazide (3 mg/kg/10 mg/kg, i.p.) using pERK antibody (**A**–**C**), pS6 (**D**–**F**), pan-pPKA substrate (**G**,**H**) and pHisH3 antibodies (**I**,**J**). Scale bars 100 μm. N = 10–12. Data are means +/− S.E.M. Statistical analysis was performed using one-way ANOVA and Dunnett’s mupltiple comparisons post-test (****P < 0.0001; **P < 0.01; *P < 0.05).

Interestingly, a transition from pERK in SPNs in response to acute/subchronic L-DOPA to pERK in CINs after long-term L-DOPA treatment (7 weeks) has been described^32^. In order to test the hypothesis that the D5 receptor mediates this activation, we examined pERK immunohistochemically in slices derived from dyskinetic mice that were perfused 30 min after the last dose of 7 weeks treatment with L-DOPA. The percentage of CINs expressing pERK was significantly reduced in the D5 KO mice (by 58% p < 0.0001, 52.3 +/− 7.7 of CINS in WT mice while only 22.1% +/− 5.8% of Cins in the D5KO were pERK positive) while the number of total pERK-positive cells (non-CIN) which by a large majority represent the spiny projection neurons (SPNs) was not altered (Fig. 4A–C). To determine whether these effects are specific to the striatum we also counted pERK-positive cells in the subthalamic nucleus (STN) which expresses the D5 receptor, in addition to D1, D2, D3 receptors^36^. As a result, we did not detect significant differences between wild type and D5 KO animals, indeed the level of pERK-positive cells was very low (1.5–2.5 cells per STN) and pERK was not translocated into the nucleus. Furthermore, it was impossible to distinguish between hemispheres indicating that pERK is not significantly activated in a L-DOPA dependent manner in this brain region (Suppl. Fig. S2).

We also analyzed pERK expression after an acute injection of L-DOPA (3 mg/kg), using pERK and ChAT antibodies. As a result, only a very small percentage of CINs (in WT 2.2 +/− 0.9% and in KO 1.0 +/−0.7%) were pERK-positive (Suppl. Fig. S3A,B), confirming findings by others^32^. Thus, after acute stimulation with L-DOPA a difference in ERK activation cannot be detected which may be due to the low activation status of CINS at this early point. The number of total pERK-positive cells SPNs was not altered between the genotypes (Suppl. Fig. S3C).

In addition to ERK activation, phosphorylation of the ribosomal protein S6 was described by others to correlate with CIN activity, outside the context of LIDs^37^. Therefore, we also assessed S6 phosphorylation status and found a 12% reduction in the numbers of pS6-positive CINs (p < 0.05) while the total number of pS6 positive cells in the DL striatum was not altered in the KO mice (Fig. 4D–F). In addition, we examined members of the cAMP-PKA cascade, using antibodies that recognize the phosphorylated PKA substrate sequence (pan pPKA substrate) and of pS10 of HisH3. Both markers were not changed in the CINs nor in the SPNs (Fig. 4G–J and Suppl. Fig. 4A,B) in slices of KO mice.

**Figure 5:**
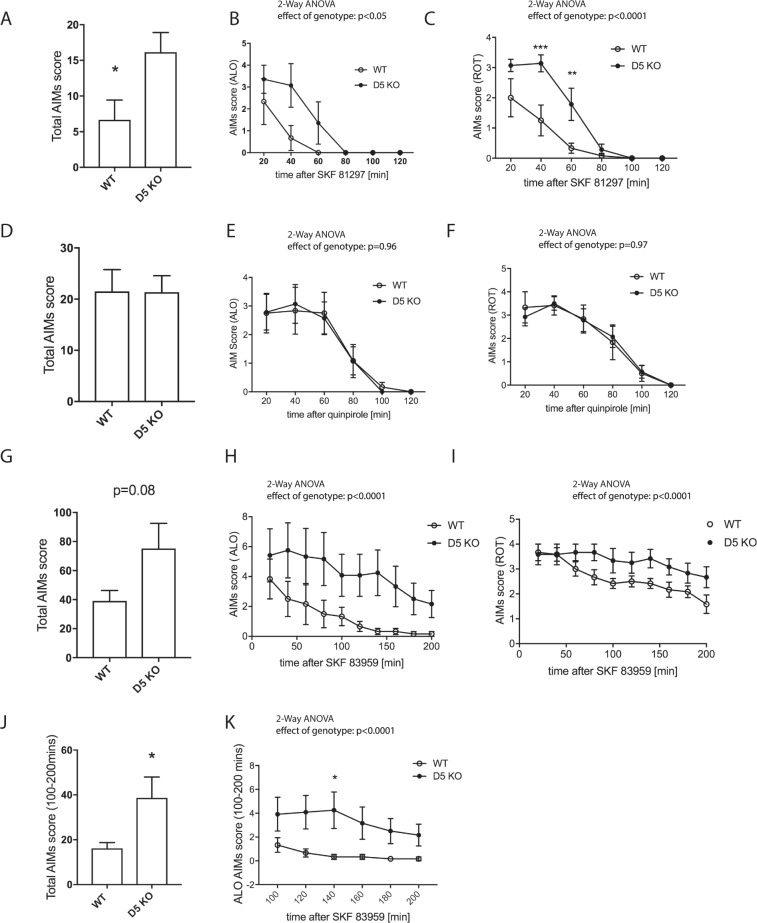
Effect of D1 and D2 agonists on LID. D5 KO and WT littermates were treated with L-DOPA/Benserazide for 7 days. On day 8 SKF81297 (2 mg/kg, i.p.) was injected instead of L-DOPA and total AIMs, ALO and ROT scores assessed (**A–C**). After one day of L-DOPA treatment, on day 10 animals received quinpirole (0.5 mg/kg, i.p.) alone, and total AIMs, ALO and ROT scores were assessed (**D–F**). N = 7. On day 40 animals received SKF83959 (0.2 mg/kg, i.p.), and total AIMs, ALO and ROT scores were assessed (**G–K**). N = 6. Graphs show mean values +/− SEM. Statistical analysis was performed using Student’s t-test (A (p < 0.05), D (p = 0.98), G (p = 0.08), J (p < 0.05) and 2-way ANOVA (effect of genotype: (**B**): F(1,66) = 5.75, p < 0.05; (**C**): F(1,66) = 19.27, p < 0.0001; (**E**): F(1,66) = 0.002, p = 0.96; (**F**): F(1,66) = 0.0015, p = 0.97; (**H**): F(1,100) = 28.31, p < 0.0001; (**I**): F(1,100) = 20.73, p < 0.0001), (**K**) F(1,60) = 27.24, p < 0.0001). Sidak’s multiple comparisons post-test (***P < 0.001; **P < 0.01).

Non-specific antagonism at muscarinic receptors was shown to reduce LID^38^, presumably due to its action on postsynaptic M1 and M4 receptors expressed on SPNs. We wanted to assess whether the muscarinic antagonist dicyclomine has a differential effect on the genotypes. After the low dose L-DOPA test (Suppl. Fig. 1A), we gave another 2 daily doses of L-DOPA 3 mg/kg, followed by co-injection of dicyclomine (45 mg/kg) with low-dose of L-DOPA. As a result, we found that dicyclomine plus L-DOPA did not affect total dyskinesia scores induced by L-DOPA alone in both genotypes (Suppl. Fig. 1A,B). With dicylomine, ALO scores remained significantly higher in the KO (Suppl. Fig. 1D), while the total LID score did not show a significant difference, albeit the trend remained (Suppl. Fig. 1B). Pre-injection of lower dicyclomine doses (20 mg/kg and 30 mg/kg) administered 30 min before or at a dose of 20 mg/kg co-injected with L-DOPA (3 mg/mg) did not reduce LID scores. We also tested the dyskinetic response of the mice to D1-like agonist SKF 81297 (2 mg/kg) and quinpirole (0.5 mg/kg), a D2-Type agonist in a separate cohort of mice. With SKF 81297, the difference in total AIMS score between genotypes persisted (Fig. 5A, p < 0.05). We observed a shorter duration of dyskinetic symptoms with SKF 81297 than with L-DOPA or quinpirole. The genotype difference was mainly due to a significantly higher rotational score in the KO mice (Fig. 5C, p < 0.0001), but an effect was also observed in the ALO score (Fig. 5B, p < 0.05). With quinpirole, the difference between genotypes was abolished in total and ALO dyskinesia as well as rotational scores (Fig. 5D–F). A different D1-type agonist, SKF 83959 (0.2 mg/kg, dosing based on^39,40^), which has by some groups been attributed to more specifically activate D5R and PLC signaling^41,42^, was tested. As a result, we found that effect of in ALO and ROT scores between genotypes persisted (effect of genotype p < 0.0001), however at individual time points no significant difference between genotypes was observed. Total AIMs were not significantly higher in the D5 KO (Fig. 5G, p = 0.08), albeit a clear trend was still detectable. The rotational effect of this agonist lasted longer than that of SKF 81297. For example, after 200 minutes, all animals were still rotating while rotation was completely abolished after 100 minutes for SKF81297-treated mice (Fig. 5C versus 5I). If results are plotted for the last part of the monitoring period (from 100 min onwards), total AIM and ALO scores are enhanced in the D5 KO (Fig. 5J,K).

This prolonged behavioral effect can be explained by the fact that SKF83959 does not strongly induce recruitment of arrestin or D1 receptor internalization^43^.

## Discussion

The important role of the D1 receptor in PD-linked akinesia and LID has been well studied but not much is known about the D5 receptor. In rodents, there is increased recruitment of D1R to the plasma membrane of SPNs^44^, as well as hyper-sensitized signaling mediated by increased adenylyl cyclase 5 activity^45^, Gα_olf_ levels^46^ or by non-canonical activation of the Ras-ERK pathway^24,25^ in direct SPNs. The fact that D5 KO mice do not exhibit altered locomotive behavior or motor learning^17^ did not put the D5 receptor in an obvious position to be investigated in LID. However, attenuated locomotor response to acute cocaine in D5 KO mice was reported^18^, as well as diminished locomotor and grooming stimulated responses to direct D1R/D5R agonists^17,32,47^. Our study shows that there is a slight but significant upregulation of the D5 receptor transcript during LID but not after DA depletion alone, hinting towards a possible involvement in chronic L-DOPA-induced network dysregulation. Since D5 KO cannot upregulate D5 levels and exhibit more pronounced dyskinesia, one may speculate that this upregulation in WT mice may be part of a response that is induced to counteract LID.

The D5 receptor is primarily expressed in CINs (88% of CINS) and to a much lesser extent in D2 SPNs (<20% of iSPNs)^13^. Interestingly, in the rotarod paradigm, while 6-OHDA lesioned mice D5 KO mice behaved equally to WT mice, when challenged with L-DOPA they exhibited a deficiency. We excluded that these findings are due enhanced sensitivity to the neurotoxin, since TH levels and contralateral paw movement in the cylinder test did not differ between genotypes. The rotarod test was performed two weeks after the first L-DOPA injection when AIMS are well developed and D5 KO exhibit enhanced LID scores. Therefore, we conclude that the worsened performance on the rotarod may be caused by enhanced dyskinesia impairing correct behavior on the rotarod.

The D1-type agonist SKF 83959 has, by some groups, been attributed to more specifically activate D5R and PLC signaling^41,42^. In rats, it was shown to attenuate LID when chronically administered with L-DOPA^48^. If the agonist specifically targets D5, we expected to it to preferentially act upon the WT mice and possibly reduce its LID, thereby enhancing the behavioral difference between genotypes. This was partially the case, specifically during later time points and may therefore be more related to aspects of receptor homeostasis than activation. Because of the conflicting literature regarding this agonist^41,42^ and the treatment regimen whereby SKF83959 alone was administered to dyskinetic mice, we cannot draw a conclusive statement that would confirm the LID-reducing effect of this agonist or its bias towards either D1-type receptor.

It was very important to assess if CIN activity markers are altered in the D5 KO mice. The sensitivity of dSPNs during LID is well established and leads to elevated signaling within the MAPK and cAMP/PKA pathways^24,28^. In particular, the amount of pERK positive cells in D1 SPNs in the striatum correlates with dyskinesia severity^28^, while a correlation is not necessary for PKA substrate phosphorylation^49,50^. Ding *et al*. showed that repeated L-DOPA administration leads to ERK phosphorylation in CINs which underlies higher basal firing and potentiated excitatory responses to DA in CINs concomitant with LID expression^32^.

The D5 receptor is the most probable candidate to mediate this ERK phosphorylation as the major Gα_s_-coupled DA receptor expressed in CINs^14^. Expectedly, a reduction in pERK in CINs was observed in the D5 KO mice. The finding that, despite the lack of D5 receptor, we still detected pERK-positive CINs in response to L-DOPA is most probably due to the 17% of CINs co-expressing D1 receptor^14,51^.

The pERK signal in dSPNs is not altered in D5 KO. One could also have expected an indirect effect on these neurons, possibly mediated via reduced M4 signaling. However, it was shown by others that after prolonged L-DOPA (7 weeks), only approximately 10% of SPNs are pERK-positive while the number pERK-positive CINs is strongly enhanced, implying that at this stage pERK in dSPNs may not be the major driver of LID^32^. Our study indicates that the phosphorylation status of ERK in CINs is not correlative with LID since ERK phosphorylation in CINs was reduced while dyskinesia was enhanced in D5 KO mice.

Another biochemical marker of CIN activity is phosphorylated ribosomal protein S6, a downstream member of the mTor signaling pathway^37^. D5 KO mice exhibit reduced levels of this marker, again suggesting that CINs in the KO mice are less active. In the unlesioned hemisphere, no pERK is detectable after L-DOPA for both genotypes while low levels of pS6, pPKA substrate, and pHisH3 are detected but not altered in the D5 KO mice. In summary, pERK and pS6 but not the PKA cascade pathway were found to be altered in CINs during the expression of LID.

Our study will undoubtedly add to the current and ongoing discussion on the role of CINs in LID. Modulation of SPNs by CINs is intricate, and can occur directly via pre- and post-synaptic muscarinic acetylcholine receptors (mAChRs), or indirectly via nicotinic acetylcholine receptors (nAChRs), expressed at terminals of DA neurons^52^. Although all five mAChR subtypes are expressed in the striatum, the inhibitory M4 and the activating M1 receptors are the major modulators of dSPNs, while the M1 is important for iSPNs which express the M4 at much lower levels^52–55^. The M1, M3, and M5 mAChRs are coupled to G_q/11_ proteins that activate phospholipases and mobilize intracellular Ca^2+^ while M2 and M4 mAChRs are coupled to G_i/o_ proteins and inhibit adenylyl cyclase^52^.

It is not clear if and through which receptors, CIN activity is beneficial or detrimental to the expression of dyskinesia since controversial results were published: selective ablation of striatal CINs was shown to decrease LIDs^56^. The mechanism proposed was through action of muscarinic receptors since non-selective muscarinic receptor antagonism (dicyclomine) decreased LID^32^. However, others were unable to detect such an effect using the muscarinic antagonist atropine but showed that nicotine, nicotinic agonists and antagonists reduced LID^38,57^. We also tested the general muscarinic antagonist dicyclomine in our mice: we used several doses ranging from 15 to 45 mg/kg, either co-injected with or pre-injected 30 min prior to L-DOPA. We did not detect a significant LID-lowering effect or differences in response between the genotypes which could be due to the non-specificity of dicyclomine masking a D5-specific effect.

A recent study showed, using optogenetic activation of CINs with different stimulation protocols, that both types of ACh receptors are involved, in opposing ways, in the expression of LID: Short duration pulses (1–5 ms) enhance LIDs, in a mAchR-dependent manner, while stimulation with a longer duration pulses (20 ms-1s) reduced LIDs in a nAchR-dependent manner^57^. Interestingly, pretreatment with a general nAChR antagonist mecamylamine, reversed the effect of optical stimulation and lead to enhanced dyskinesia^57^. Since nicotinic receptors were silenced during the experiment, clearly, muscarinic receptors have played a role in this response. Chemogenic activation studies have revealed that striatal CIN activation potentiates the anti-akinetic effect of L-DOPA and aggravates the D2R-mediated portion of LIDs^58^.

Focusing on specific muscarinic receptors, it was shown that enhancing postsynaptic M4 signaling via positive allosteric modulators promoted LTD, blunted LTP in dSPNs and decreased dyskinesias^31^. The M4 receptor is the most abundant striatal muscarinic receptor and is preferentially expressed in dSPNs where it is clustered near axospinous glutamatergic synapses^59^. This finding is not necessarily contradicting results with generic mACh antagonism and LID reduction, due to the different signaling modes of M1 (activating) and the M4 (inhibitory) receptors both of which are also expressed as autoreceptors.

Thus far, all CIN-specific optogenetic or chemogenic manipulations were performed to enhance activity. The only approach to investigate the outcome of CIN inactivation was their selective ablation which lead to reduced LID^56^. However, it was argued by others that a complete and chronic ablation of CINs will lead to unknown molecular as well as physiological adaptations at synapses and complicate comparisons^31^. Therefore, our results would be better compared to the outcome of more acute and/or subtle reduction in CIN activity, such as for example using optogenetic/chemogenic tools.

Interestingly, studies looking at the role of D5 in learning showed that in hippocampal neurons D5 receptor activation leads to Ach release which is significantly reduced in D5 KO mice^60^. Furthermore, M1 but not M2 receptor expression is enhanced in all regions of the hippocampal formation^60^. It is very plausible that such an adaptation also occurs in the striatum. Since activating M1 receptors are expressed postsynaptically on dSPNs, one would expect this to enhance dSPN excitability and possibly mediate the LID phenotype. A combination of upregulation of the postsynaptic M1 and reduced activation of the M4 receptors might be at play. The finding that dyskinetic D5 KO mice challenged with D1 but not D2 agonist maintain the enhanced dyskinesia phenotype, further argues for an important effect of D5 KO on dSPNs.

Interestingly, in D5 KO mice, LTD, a specific form of corticostriatal plasticity, is abolished^61^. Reduction in LTD is also consistent with elevated LID^31^.

Crossing D5 KO mice with bacTrap mice where ribosomes of CINs are GFP labeled, would enable cell specific RNA purification/sequencing and the determination of changes in expression of the various receptor involved (e.g. M1, M4, D2). Similarly, future studies using the D5 KO mice crossed to ChAT-GFP and/or Drd1a-tdTomoato labeled mice will allow electrophysiological studies to determine changes in acetylcholine release and the synaptic plasticity of dSPNs in the D5 KO mice which would put the above stated hypotheses to the test.

## Conclusion

Our study is the first to investigate the effect of genetic inactivation of the D5 receptor in a mouse model of PD. D5 KO mice, when rendered parkinsonian by unilateral 6-OHDA injection, showed reduced motor ability on the rotarod in response to L-DOPA, altered expression of activity markers of CINs and enhanced expression of LID. In the context of the discussion of the role of CINs in LID expression, our findings add a novel previously overlooked important player that modulates cholinergic activity and most probably leads to adaptions within the homeostatic network of DA and ACh receptors.

## Methods

### Animals

The generation of the dopamine D5 receptor KO mice was described^17^. The mice were backcrossed with C57BL/6 mice for at least 10 generations before testing. Mouse genotypes were confirmed by PCR. Animals that underwent 6-OHDA lesions were of 25–30 g in weight (4–6 months old). The mice were maintained in a 12 h light/dark cycle, with access to food/water ad libitum. Animal procedures were in accordance with the National Institutes of Health (NIH) guidelines and approved by City College New York’s institutional animal care and use committee (IACUC).

### Reagents and antibodies

SKF 81297 and quinpirole were from Tocris (Minneapolis, MN, USA), dicyclomine, 6-OHDA-HCl, L-DOPA (3-(3,4-Dihydroxyphenyl-2,5,6-d_3_)-L-alanine), desipramine, pargyline and benserazide were purchased from Sigma-Aldrich (St. Louis, MO, USA). Anti-tyrosine hydroxylase (RRID: AB_390204) and anti-Choline Acetyltransferase (RRID:AB_2079751) and anti-pS10H3 (RRID:AB_1977177) were purchased from Millipore. Anti-phospho-Thr202/Tyr204–Erk1/2 (RRID:AB_331646), and phospho-(Ser/Thr) PKA substrate (RRID:AB_331817), a-DARPP-32 (RRID:AB_823479).

And anti pS6 ribosomal protein (RRID:AB_916156) were from Cell Signaling Technology. Various Alexa Fluor antibodies were used at a 1:500 dilution for immunohistochemistry (Invitrogen).

### 6-OHDA lesions and L-DOPA treatment

Mice were injected with desipramine/pargyline (25 mg/kg/5 mg/kg; i.p.) before the start of the surgery. Unilateral 6-OHDA injections into the medial forbrain bundle were performed according to a well-established method^35^. Mice were anesthetized with a mixture of ketamine (80 mg/kg) and xylazine (12 mg/kg); the local anesthetic Bupivacaine (Marcaine) was subcutaneously injected near the surgery site. 6-OHDA-HCl (3.0 mg/ml; Sigma-Aldrich) was dissolved in a solution containing 0.2 g/l ascorbic acid. The medial forebrain bundle (MFB) was targeted by a single injection of 3 μg of 6-OHDA at coordinates: AP: −1.2 mm and ML: −1.1 mm and DV: −5.0 mm from dura. Each injection was performed with a Hamilton needle (33 gauge) connected to a syringe micropump (WPI) by a polyethylene catheter, at a slow rate of 0.3 μl/min to minimize tissue damage. After the injection, the needle was left in place for an additional 4 min before being slowly retracted. After surgery, animals were kept warm with a heated mat and observed three times daily. If necessary, animals received injections of 4% sucrose (10 ml/kg, s.c.) and saline (10 ml/kg, i.p.) as well as hydrogel pouches to avoid dehydration and hi-fat chow to reduce weight loss. Mice were allowed to recover for 3 weeks before behavioral evaluation and drug treatment. Lesions were assessed biochemically at the end of experiments by determining the striatal levels of tyrosine hydroxylase (TH) using immunohistochemistry. Only animals with a TH-depletion of 90% of the striatal area when compared to unlesioned side were included in the analyses. If not indicated otherwise, treatment of L-DOPA (3 mg/kg)/Benserazide (10 mg/kg) was administered i.p. over seven consecutive days, starting 3 weeks after the lesion. Drugs were dissolved in physiological saline and administered at the volume of 10 ml/kg body weight.

### Immunohistochemistry

Thirty minutes after the last i.p. injection, animals were anesthetized with pentobarbital/ phenytoin and transcardially perfused with 50 ml of 4% paraformaldehyde (PFA) in PBS. Brains were incubated overnight in 30% sucrose and sliced at a thickness of 30 μM. Incubations with antibodies and analysis were performed as described elsewhere^49^. Sections were mounted using Prolong Gold (Invitrogen). Confocal microscopy was performed using Zeiss LSM 710 (for most figs.) and 880 (for Suppl. Figs. S2,3) laser-scanning confocal microscopes using the same adjustments for all the sections in a given experiment. For all immunohistochemical analysis, cells were counted manually or with NIH Image J (in 425 × 425 μm confocal images) and averaged from 3 slices per animal (unlesioned and lesioned sides under blind conditions concerning the mouse genotype and treatment). TH immunofluorescence intensity was quantified in striata with NIH Image J, and the data represented as mean gray levels above background value.

### RNA extraction and real time PCR

Brain tissue from the PFC, hippocampus and striatum was homogenized and extracted using TRIZOL (Life Technologies). 5 to 10 µg of total RNA was treated with TURBO DNAse (Ambion) and cDNA was synthesized using 1–2 µg of DNA-free RNA and the High-capacity cDNA reverse transcription kit with random primers (Applied Biosystems). Relative mRNA levels were quantified on a Realplex PCR machine (Eppendorf) using Taqman gene expression assays for Drd5 (Mm04210376_s1). Relative expression was determined with the ΔΔCt method (^62^, PMID: 11846609) using GAPDH mRNA (Mm99999915_g1).

### Cylinder test

The anti-akinetic effect of L-DOPA was assessed using the cylinder test of forelimb paw placement. Mice were placed in a glass cylinder (10 cm wide × 14 cm high) and recorded for 4 minutes. The number of supporting paw placements performed independently with the left and right paw were counted. The limb use asymmetry score was calculated by expressing the number of wall contacts performed with the forelimb contralateral to lesion as a percentage of total wall contacts^63^. For tests performed with L-DOPA, the mice were tested 1 hour after first L-DOPA dosage.

### Rotarod

Assessment of motor coordination was carried out by rotarod test. Mice were tested before 6-OHDA lesion, after lesion, and after L-DOPA treatment. On the day of testing, mice were trained for 1 minute by placing on an immobile rod. During this training session, the mice were placed back onto the rod if they fell. Testing consisted of placing the mice on an accelerating rotarod from 4 to 40 rpm for 5 minutes. Latency to fall was recorded and testing was repeated for five consecutive trials, 20 minutes apart. Testing for L-DOPA treatment was done 90 minutes after L-DOPA injection.

### *A*bnormal *i*nvoluntary *m*ovements (AIMs)

AIMs were evaluated by an observer blind to the genotype, starting 20 min after administration of L-DOPA/Benserazide (+/− additional drugs as described). Abnormal movements, clearly distinct from natural or stereotypic behaviors (i.e., grooming or sniffing), were classified into four different subtypes as described^64^: locomotive (tight contralateral turns) (ROT), axial (contralateral dystonic posture of the neck and upper body), limb (jerky, fluttering movements of the limb contralateral to the lesion), and orofacial (vacuous jaw movements, tongue protrusions) AIMs. Each subtype was scored on a severity scale from 0 to 4: 0, absent; 1, occasional; 2, frequent; 3, continuous; 4, continuous and not interruptible by external stimuli. The total AIMs score corresponded to the sum of individual scores for each AIM subtype. A composite score was obtained by the addition of scores for axial, limb, and orofacial AIMs (ALO score). The ALO score is considered to more closely reflect the human dyskinetic behavior than the locomotive AIMs score (LOC score).

### Statistical analysis

Behavioral data were analyzed by two-way analyses of variance (ANOVA) followed by Sidak’s *post hoc* test or Student’s unpaired t-test. Immunohistochemistry data and qPCR were evaluated by Student’s paired and unpaired *t*-test. Analysis was performed with GraphPad Prism 6. Data are expressed as the mean ± standard error of the mean (SEM) and the significance level was set to *P* < 0.05.

## Supplementary information

Supplementaryinformation
