# Publisher Correction: The Dopamine D5 receptor contributes to activation of cholinergic interneurons during L-DOPA induced dyskinesia

**DOI:** 10.1038/s41598-020-61832-3

**Published:** 2020-03-13

**Authors:** Julia Castello, Marisol Cortés, Lauren Malave, Andreas Kottmann, David R. Sibley, Eitan Friedman, Heike Rebholz

**Affiliations:** 1 Department of Molecular, Cellular & Biomedical Sciences, CUNY School of Medicine, New York, NY USA; 2 Ph.D. Programs in Biochemistry and Biology, The Graduate Center, CUNY, New York, USA; 3 Molecular Neuropharmacology Section, National Institute of Neurologic Disorders and Stroke, Intramural Research Program, National Institutes of Health, Bethesda, Maryland USA; 4 Institut de Psychiatrie et Neurosciences de Paris (IPNP), UMR_S1266, INSERM, Université de Paris, 102-108 rue de la Santé, F-75014 Paris, France; 5 GHU PARIS psychiatrie et neurosciences, Paris, France; 6 Danube Private University (DPU), Krems, Austria

Correction to: *Scientific Reports* 10.1038/s41598-020-59011-5, published online 13 February 2020

This Article contains an error in the Introduction section.

“Hallmarks of the disorder are motor symptoms such as tremor, rigidity, bradykinesia and gait disturbances^2^, which are a result of the selective degeneration of dopaminergic neurons within the substantia nigra pars compacta and thus not by but of dopamine depletion^3^.”

should read:

“Hallmarks of the disorder are motor symptoms such as tremor, rigidity, bradykinesia and gait disturbances^2^, which are a result of the selective degeneration of dopaminergic neurons within the substantia nigra pars compacta and thus of dopamine depletion^3^.”

