# Supplementary material for: The Dopamine D5 receptor contributes to activation of cholinergic interneurons during L-DOPA induced dyskinesia
Source: Sci Rep. 2020 Feb 13;10:2542. doi: 10.1038/s41598-020-59011-5 (PMC7018760; doi:10.1038/s41598-020-59011-5)
Supplement: Supplementary file 1 — Supplementaryinformation [file 41598_2020_59011_MOESM1_ESM.pdf]

# The Dopamine D5 receptor contributes to activation of cholinergic interneurons during L-DOPA induced dyskinesia

Julia Castello[*ΦΣ], Marisol Cortés[*Σ], Lauren Malave[*], Andreas Kottmann[*], David R Sibley[Ω], Eitan Friedman[*Φ] and Heike Rebholz[*ΔΞΨ]

[*]Department of Molecular, Cellular & Biomedical Sciences, CUNY School of Medicine, New York, NY USA; [Φ]Ph.D. Programs in Biochemistry and Biology, The Graduate Center, CUNY, New York, USA; [Ω] Molecular Neuropharmacology Section, National Institute of Neurologic Disorders and Stroke, Intramural Research Program, National Institutes of Health, Bethesda, Maryland, USA; [Δ] Institut de Psychiatrie et Neuroscience de Paris (IPNP-INSERM), GHU PARIS psychiatrie et neurosciences, [Ξ]Danube Private University (DPU), Krems, Austria

[Ψ] **Corresponding Author:** Heike Rebholz, heikerebholz@gmail.com

[Σ] these authors contributed equally

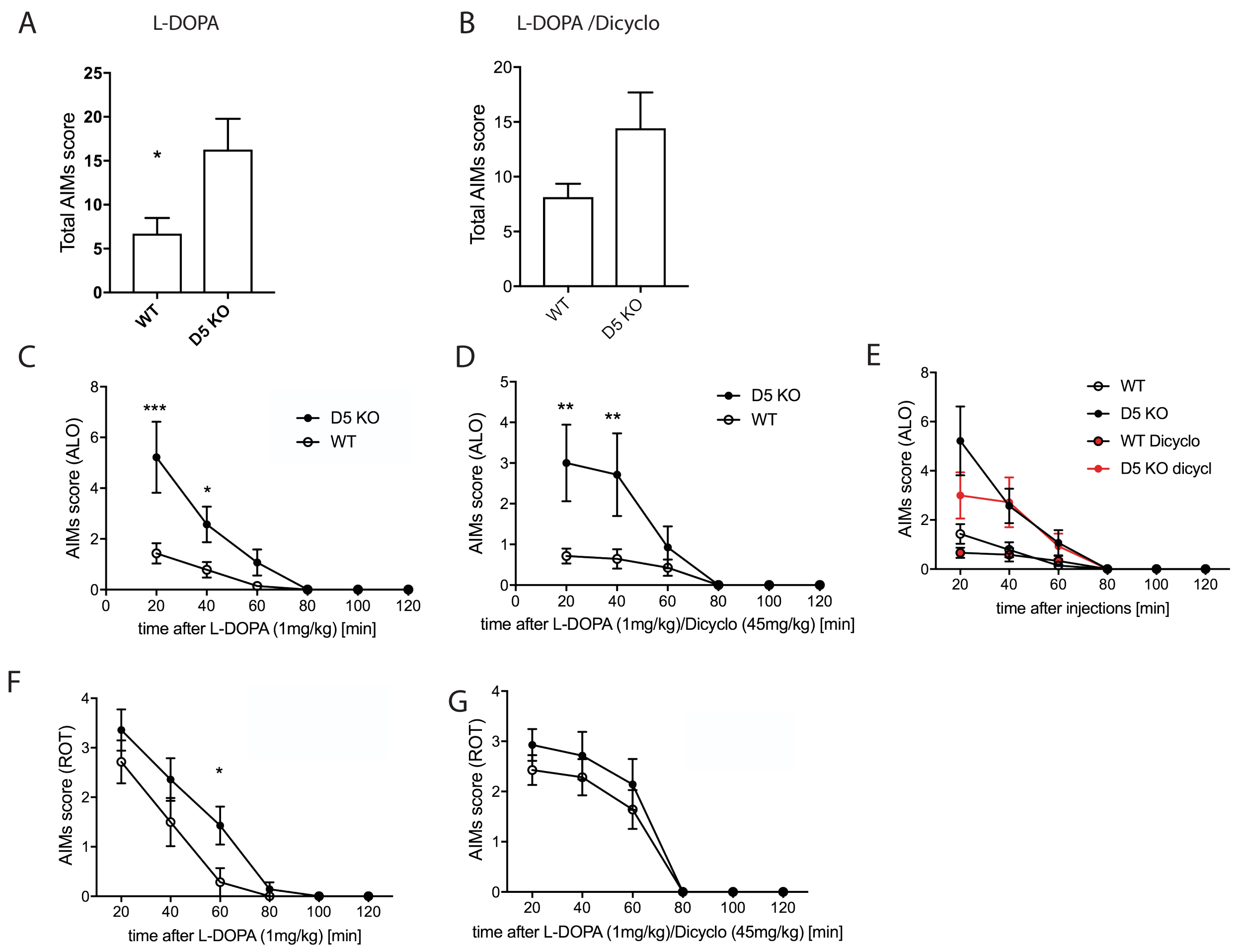


Fig. S1 Effect of low dose L-DOPA (1mg/kg) and dicyclomine on LID
Mice were rendered dyskinetic with L-DOPA/ Benserazide (3mg/kg/ 10 mg/kg) and on day 34 were tested with low dose L-DOPA (1mg/kg) (A,C,F). Thereafter, L-DOPA (3mg/kg) was injected for 2 more days, after which L-DOPA (1mg/kg) and the M1 muscarinic receptor antagonist dicyclomine (45 mg/kg) were co-injected with L-DOPA and LID was assessed (B,D,E,G). N=7. Graphs show mean values +/- SEM. Statistical analysis was performed using Student's t-test for (A ($p<0.05$), B ($p=0.1$) and 2-way ANOVA (Effect of genotype C: $F(1,72)=14.1$, $p=0.0003$, D: $F(1,72)=10.15$, $p<0.005$; F: $F(1,72)=7.46$, $p<0.01$; G: $F(1,72)=2.14$). Sidak's multiple comparisons post-test (***, $p<0.001$;**, $p<0.01$, *,$p<0.05$).

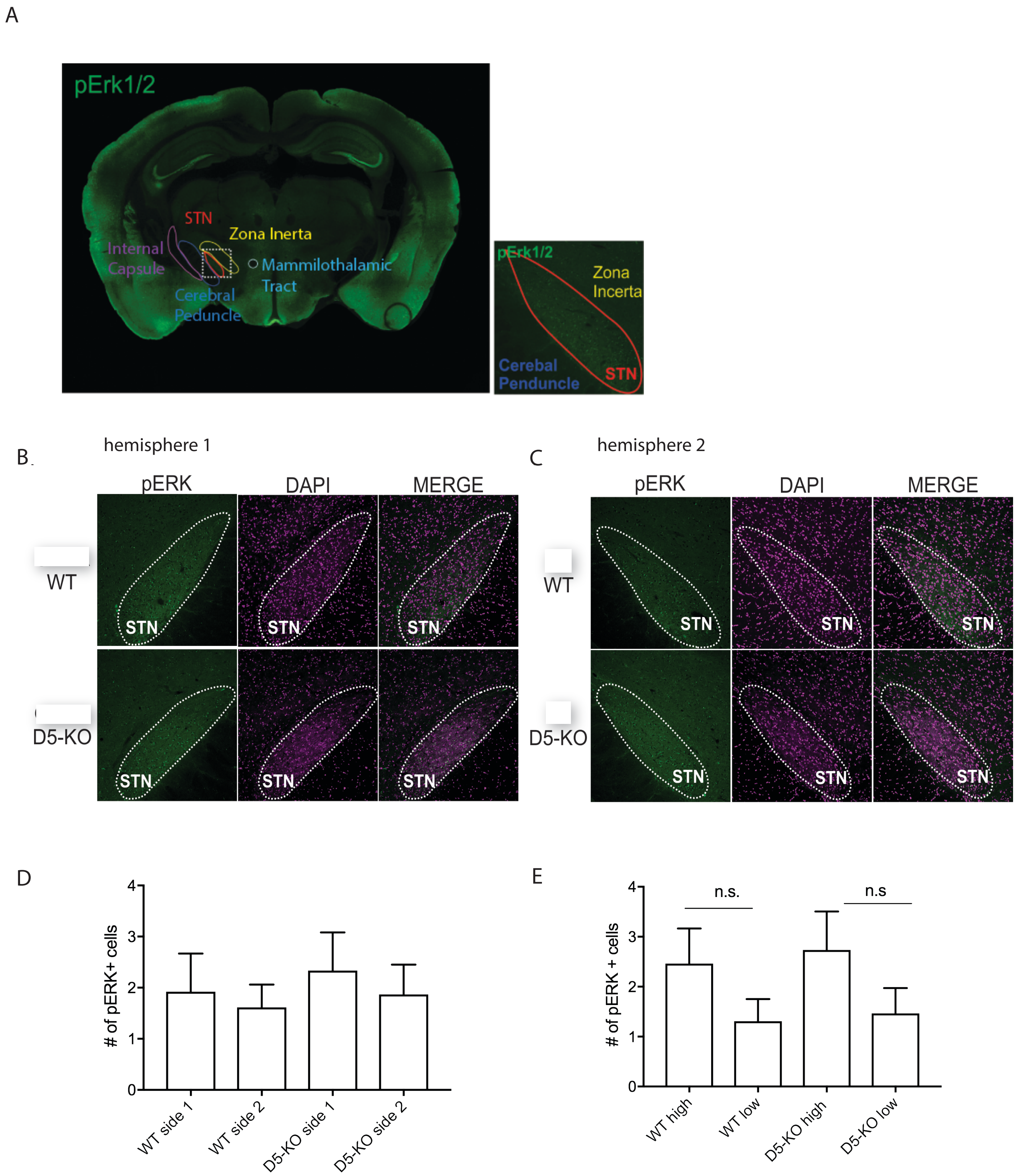


Fig. S2 Immunohistochemical analysis of pERK in the STN.
Scheme of the localisation of the STN in mouse coronal sections (A). Coronal slices comprising the midbrain region from WT and Drd5-KO mice were used for IHC with pERK and ChAT antibodies after chronic L-DOPA/Benserazide (3mg/kg/10mg/kg, i.p.) (B,C). Quantification: For analysis, slices were randomaly assigned a hemispere (D), or higher values of the pairs (left or right sides) were compounded and plotted (E). None of these anlyses resulted in a signifcant difference between hemispheres or genotypes. Graphs show mean values +/- SEM. Statistical analysis was performed using 2-way ANOVA (N=7).

Suppl. fig. S2

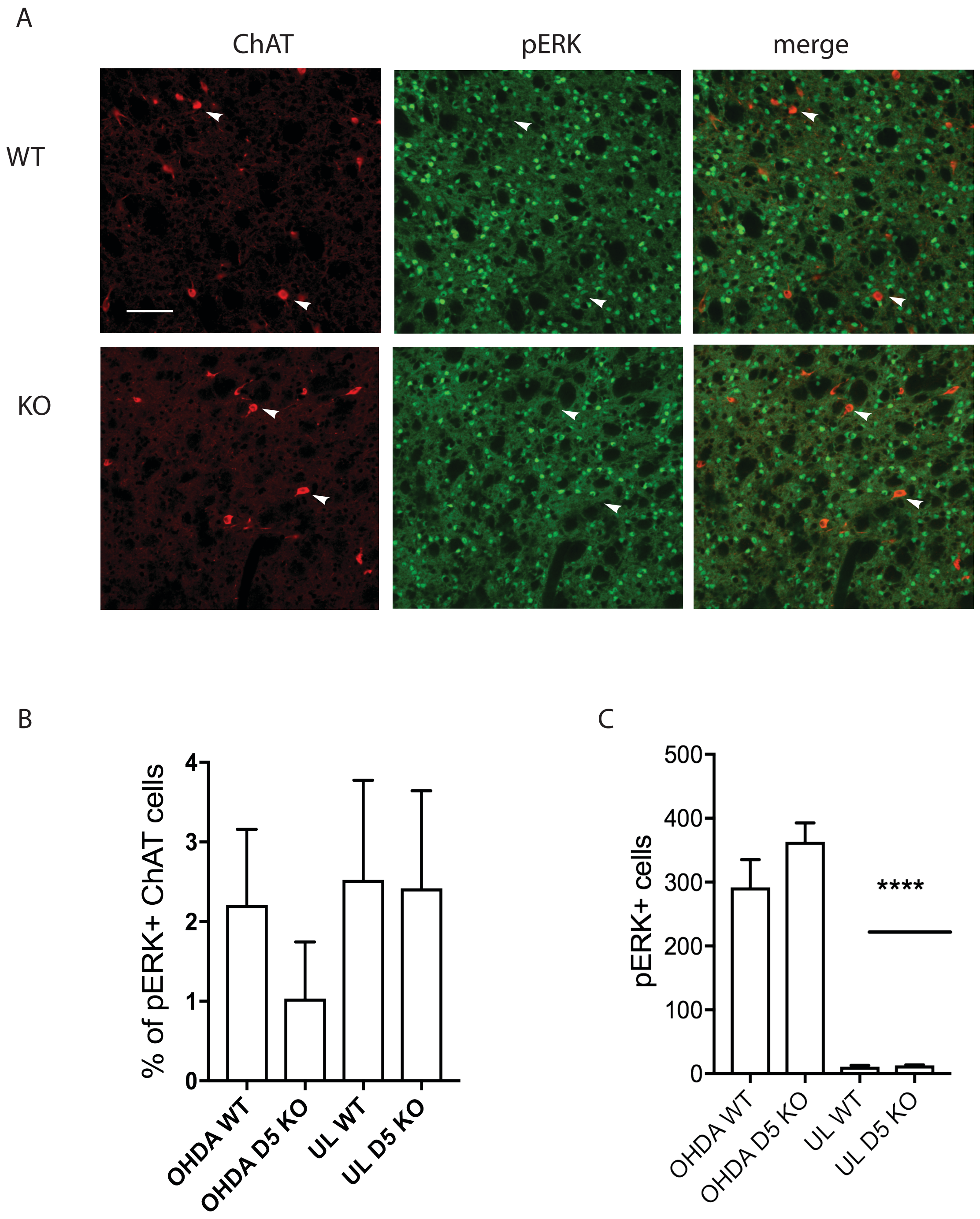


Fig. S3. Immunohistochemical analysis of pERK after acute L-DOPA.
Coronal striatal slices from WT and Drd5-KO mice acutely treated with L-DOPA/Benserazide (3mg/kg/10mg/kg, i.p.), were used for IHC with pERK and ChAT antibodies (A). Quantification thereof (B, C). Scale bar 100 µm, arrows point to CINs. Graphs show mean values +/- SEM. Statistical analysis was performed using 2-way ANOVA,****, P<0.0001 (N=6).

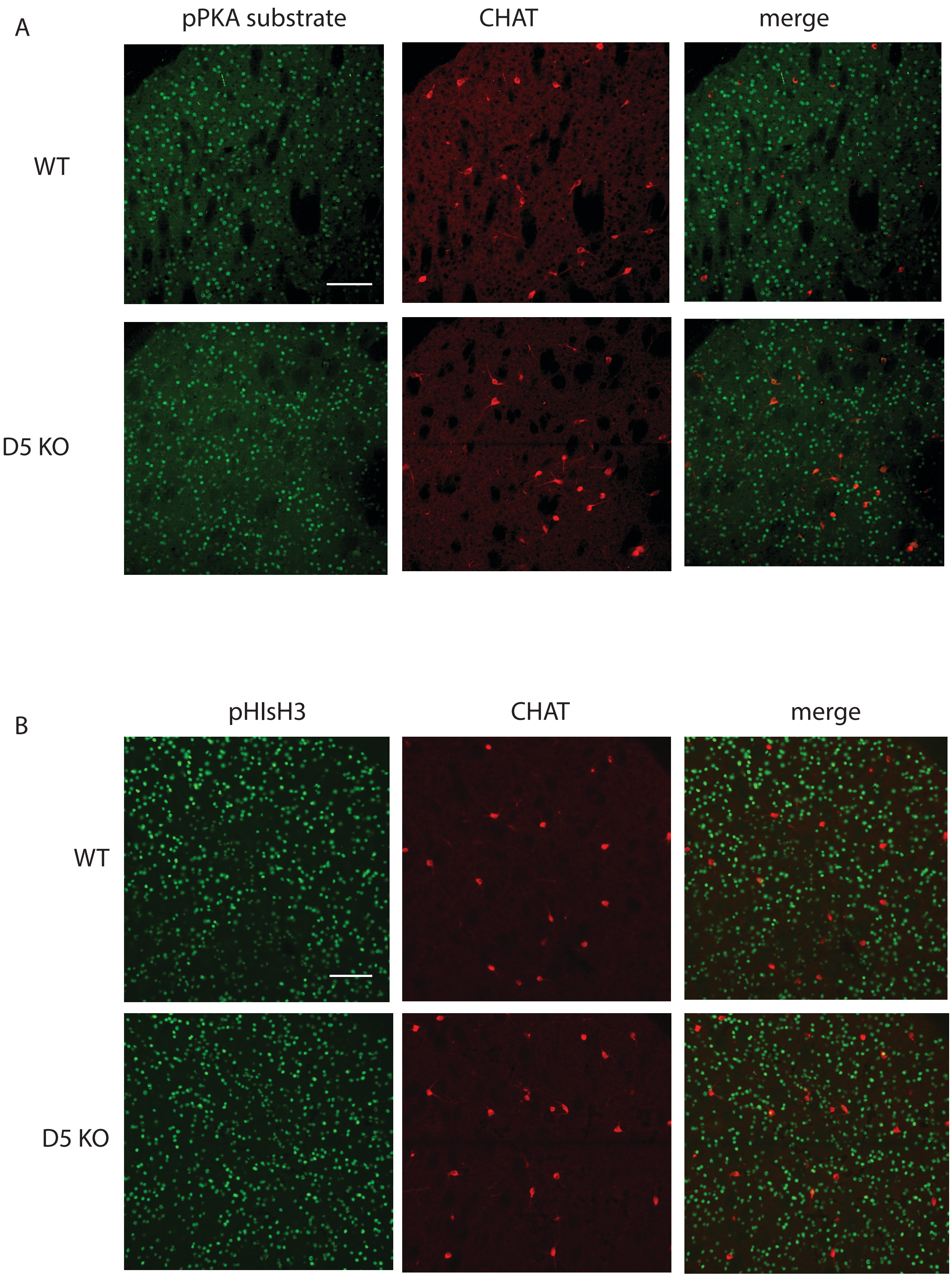


Fig. S4. Immunohistochemical analysis of coronal dorsolateral striatal slices from WT and Drd5-KO mice after chronic L-DOPA/Benserazide (3mg/kg/10mg/kg, i.p.) using pan-pPKA substrate (A) and pHisH3 antibodies (B). Scale bars 100 µm.

Suppl. fig. S4
